# Bioactivity-guided isolation and molecular modeling of the anti-inflammatory constituents from the leaves of *Duranta erecta* Linn.

**DOI:** 10.1186/s12906-025-04764-7

**Published:** 2025-01-28

**Authors:** Marina Sobhy, Farid N. Kirollos, Sameh F. AbouZid, Nasser S. M. Ismail, Riham A. El-Shiekh, Essam Abdel-Sattar

**Affiliations:** 1 Pharmacognosy Department, Faculty of Pharmacy, Heliopolis University, Cairo, Egypt; 2 Pharmacognosy Department, Faculty of Pharmacy, Cairo University, Cairo, Egypt; 3 Department of Pharmacognosy, Faculty of Pharmacy, Beni-Suef University, Beni-Suef, 62514 Egypt; 4 Pharmaceutical Chemistry Department, Faculty of Pharmacy, Future University in Egypt (FUE), Cairo, 11835 Egypt

**Keywords:** *Duranta erecta* Linn., Bioactivity-guided isolation, Molecular docking, Molecular dynamics, Modelling ADME properties, Anti-inflammatory

## Abstract

*Duranta erecta* Linn. belongs to the Verbenaceae family and is primarily found in subtropical, tropical, and temperate climates. The plant has been reported to contain a variety of phytoconstituents, including iridoid glycosides, flavonoids, flavonoid glycosides, alkaloids, phenolics, tannins, terpenoids, steroids, and saponins. In this investigation, a bioactivity-guided isolation was used to isolate cyclooxygenases (COXs) and lipoxygenase (LOX)-inhibiting compounds from the leaves of *Duranta erecta* Linn. Duranterectoside A (**1**), lamiide (**2**), and apigenin 4’,7-dimethyl ether (**3**) were identified employing spectroscopic methods (including ESI-MS, ^1^H-NMR, and ^13^C-NMR), and by comparing with existing literature data. This is the first report of metabolitrs from *D. erecta* inhibiting both LOX and COX enzymes. Furthermore, the isolated compounds were analyzed using computerized virtual screening, which enabled the modelling ADME characteristics, molecular docking, and dynamics simulation. The results demonstrated that compound **1** had greater docking scores than the docked lead compounds. Overall, the data reported in this study add to our understanding of the pharmacological properties of the examined plant and Duranterectoside A (**1**) and pave the way for future research and investigation in inflammation and drug discovery.

**Supplementary Information:**

The online version contains supplementary material available at 10.1186/s12906-025-04764-7.

## Introduction

Unresolved inflammation can lead to a variety of pathological disorders, including inflammatory bowel disease, atherosclerosis, arthritis, cancer development, and bronchial asthma [1]. In these conditions, one of the key mechanisms causing inflammation is the arachidonic acid route, which includes two separate metabolic pathways. The cyclooxygenase (COX) pathway, which involves the synthesis of prostanoids such as prostaglandins, prostacyclins, and thromboxanes, catalyzed by cyclooxygenase-1 (COX-1) and cyclooxygenase-2 (COX-2) enzymes. The lipoxygenase (LOX) pathway, on the other hand, produces leukotrienes (LTs) and hydroperoxy fatty acids, which are catalyzed by enzymes such as 5-lipoxygenase (5-LOX), 12-lipoxygenase (12-LOX), and 15-lipoxygenase [2]. Non-steroidal anti-inflammatory medicines (NSAIDs) are at the forefront of mainstream medicine’s approach to inflammation. These medicines efficiently reduce inflammatory mediator production by reducing the activities of cyclooxygenase-1 (COX-1) and cyclooxygenase-2 (COX-2). However, COX-1 inhibitors frequently cause side effects, such as gastrointestinal discomfort and renal damage [3]. The current generation of selective COX-2 inhibitors has increased efficacy against inflammation while lowering gastrointestinal irritation. Rofecoxib (Vioxx^®^) is a well-known selective COX-2 inhibitor that was withdrawn from the market due to an elevated risk of heart attack [4].

A recent study reveals that suppressing COXs with NSAIDs may mistakenly divert the arachidonic acid route, potentially leading to an increase in the generation of pro-inflammatory leukotrienes (LTs) via the 5-lipoxygenase (5-LOX). These LTs can exacerbate the inflammatory response, counteracting the intended anti-inflammatory actions of COX inhibitors [5].

To confront this challenge directly, the synthesis of natural compounds with the ability to simultaneously inhibit both COX-2 and 5-LOX holds significant promise. By targeting both enzymes, these dual inhibitors have the potential to amplify the anti-inflammatory benefits of each while reducing adverse effects [1]. Natural products, with their diverse chemical structures, provide an abundant and useful supply of potential dual COX-2/5-LOX inhibitors, ushering in a new era in inflammation management.

*Duranta erecta* Linn. belongs to the Verbenaceae family and is primarily found as a flowering upright hanging shrub [6]. It is typically found in subtropical, tropical, and temperate regions [7]. The plant has been reported to contain a variety of phytoconstituents, including iridoid glycosides, flavonoids, flavonoid glycosides, alkaloids, phenolics, tannins, terpenoids, steroids, and saponins [8]. The therapeutic effect of *Duranta* spp. have been reported to be antibacterial, antimalarial, antioxidant, and antiviral [7]. *D. erecta* is highly valued in ethnomedicine and folklore for its anthelmintic, febrifuge, and antimalarial properties [6]. Its curative properties extend to infertility treatments, neurological diseases, and even pneumonia, solidifying its reputation as a flexible and valuable natural therapy [7].

Molecular docking is a computational analysis approach that predicts how chemical structures of different metabolites interact with specific enzymes and receptors. Furthermore, it has demonstrated high precision in screening bioactive components against a diverse spectrum of targets [9–11]. Molecular docking approaches facilitate the development of innovative therapeutic medications capable of treating a wide range of chronic disorders [12]. Molecular dynamic simulations were carried out to investigate the ligand-protein interaction in motion that contributes to their stable bound conformation and to visualize the influence of ligand binding on protein conformational changes. As a result, the anti-inflammatory activities of phytoconstituents of *D. erecta* L. were examined for the first time, with the primary components isolated utilizing a bioactivity-guided fractionation technique. The primary goal of this study is to identify potential inflammatory inhibitors using in silico analysis techniques such as molecular dynamics simulation, absorption, distribution, metabolism, and elimination (ADME) properties, and molecular docking analysis.

## Materials and methods

### General experimental procedures

COX-1 and 2 inhibition assay kits (K548-100 and K547-100) were purchased from Biovision Inc., California, USA. Sigma-Aldrich provided all additional solvents and reagents used, which were of analytical quality. Sigma-Aldrich Chemicals in Germany supplied silica gel 60 (70–230 mesh), silica gel RP-18, and precoated silica gel 60 F_*254*_ plates for chromatographic isolation. To visualize chromatograms, they were sprayed with *p*-anisaldehyde-sulfuric acid and subsequently heated to 110 °C. The absorbances were measured using a Tecan microplate reader (Infinite F50, Switzerland). The NMR spectra [^1^H-NMR (400 MHz) and ^13^C-NMR (100 MHz)] were acquired using a Bruker NMR system in CDCl_3_ and DMSO-d_6_ solvents. Chemical shifts are measured in *δ* (ppm) compared to the internal standard TMS. Sigma Chemical Company (CA, USA) supplied the reagents utilized in biological experiments.

ESI-MS in negative ion mode was conducted using a XEVO TQD triple quadrupole mass spectrometer (Waters Corporation, Milford, MA 01757, U.S.A.). The instrument is equipped with reverse phase C-18 column (ACQUITY LC - BEH, 2.1 × 50 mm column; 1.7 μm particle size). The analysis was performed under these conditions: source temperature of 150 °C, cone voltage of 30 eV, capillary voltage of 3 kV, desolvation temperature of 440 °C, cone gas flow of 50 L/h, and desolvation gas flow of 900 L/h. The gradient mobile phase comprises two eluents: eluent A (H₂O acidified with 0.1% formic acid), and eluent B (acetonitrile also acidified with 0.1% formic acid) with the following gradient: (10% B) from 0 to 2 min; (30% B) from 2 to 5 min; (70% B) from 5 to 15 min; (90% B) from 15 to 25 min; and (100% B) from 25 to 29 min at a flow rate of 0.2 mL/min.

The leaves of *D. erecta* Linn were collected from a private garden owned by Engineer Safwat Habib, located at the coordinates.

### Plant material

The leaves of *D. erecta* Linn were collected from a private garden (Obour flower) with permission from the owner Engineer Ahmed Abdo. The garden is located at the coordinates 30°12’08.0"N, 31°28’22.9"E (Cairo, Egypt, in May 2022). This collection followed the guidelines endorsed by the local gardens and adhered to the scientific collection standards of Egypt. The plant was authenticated by Mrs. Trease Labib, Senior Botanist at Mazhar Botanical Garden, Cairo, Egypt, and the voucher sample (#24-1-23) was stored in the herbarium of the Department of Pharmacognosy at Cairo University’s Faculty of Pharmacy.

### Extraction and fractionation

The dry ground leaves (945 g) were extracted with 70% aqueous ethanol (1 L x 3) at room temperature. The combined 70% ethanol extract was concentrated using a rotary evaporator. The residual material (70 g) was suspended in distilled water (250 mL) and subjected to partitioning with *n*-hexane, methylene chloride, ethyl acetate, and *n*-butanol (each 250 mL x 3), resulting in the corresponding fractions after evaporation, with a yield of 17.0, 4.0, 8.0, and 40 g, respectively.

### Chromatographic isolation of the bioactive compounds from ethyl acetate fraction (Fig. S1)

The EtOAc fraction (8 g) was chromatographed on silica gel 60 using 100% CH_2_Cl_2_, using mixtures of CH_2_Cl_2_-MeOH with increasing polarity of 5–30% as eluant. This process yielded four major fractions: Fr. **A** (0.5 g), Fr. **B** (4 g), Fr. **C** (1.5 g), and Fr. **D** (1.0 g). Fraction **B** was chromatographed on a silica gel column using CH_2_Cl_2_-MeOH (95: 5 v/v) as solvent system, resulting in the isolation of compound **1** (650 mg). Fraction **C** was purified on a silica gel column using CH_2_Cl_2_ –MeOH (85: 15 v/v) as solvent system to afford compound **2** (155 mg). Compound **3** (70 mg) was separated from fraction **D** using RP-18 column with 70% MeOH/H_2_O as solvent system.

### In-vitro anti-inflammatory assays

#### COX-1 and COX-2 inhibition assay

To prepare a stock concentration of 5 mg/mL, the extract and its constituent fractions were dissolved in 100% DMSO. In triplicate, samples were serially diluted from 0.1 µg/mL to 100 µg/mL. The inhibitory activity of COX was determined using a colorimetric approach, as previously described [13], and using the Cayman colorimetric COX (ovine) inhibitor screening test kit (Cayman Chemical Company, MI, USA) according to the manufacturer’s instructions. Celecoxib was used as a positive control to suppress COX-1 and COX-2. 10 µL of each test sample and vehicle were mixed with 20 µL of 0.1 M Tris-HCl (pH 8.0) and pre-incubated with the enzyme at 37 °C for 15 min before adding arachidonic acid. After initiating the reaction with 10 µL of 10 mM arachidonic acid, the mixture was incubated at 37 °C for another 2 min. To stop the reaction, 50 µL of 1 N HCl and saturated stannous chloride were added. The assays were run using 100 U of ovine COX-1 and COX-2. An aliquot was removed, and the generated prostanoid was quantified using spectrophotometer at 405 nm. Results were presented as IC_50_ (µg/mL) [13].

#### 5-Lipoxygenase (5-LOX) inhibitory assay

The LOX inhibitor screening assay was carried out colorimetrically, as per the manufacturer’s instructions (Cayman Chemical Company, MI, USA). The samples were analyzed in triplicate at doses of 10 and 100 µg/mL, with zileuton as the positive control. In a 96-well plate, pre-incubate 10 µL of each test sample and vehicle with 90 µL of 5-LOX enzyme. To initiate the enzymatic reaction, introduce 10 µL of 1 mM arachidonic acid and shake the plate for 5 min.

To halt the enzymatic process and promote colour formation, 100 µL of chromogen from the test kit was used. The plate was then shaken for a further 5 min before the absorbance at 490 nm was measured with a microplate reader. The absorbance measurements were used to calculate the IC_50_ values (µg/mL) for 50% inhibition of LOX activity [13].

### In silico docking

The crystal structures of COX-1, -2, and LOX complexed with lead compound were retrieved from PDB using the codes (PDB ID: 3KK6) [14], (PDB ID: 1CX2) [15] and (PDB ID: 1JNQ) [16], respectively. The proteins were minimized, and the binding site was identified where dimension for COX-1(XYZ=-32.417832 43.380899–5.631524 with radius 9.969038 Ǻ), COX-2 (XYZ = 24.157072 21.793083 16.302206 with radius 11 Ǻ), LOX (XYZ = 20.415381 3.492195 19.897532 with radius 11.5 Ǻ). Compounds **1**, **2**, and **3** were drawn in ChemDraw V.14 and saved as mol extensions for later examination in Discovery Studio Software (Accelrys Inc., www.accelrys.com). The compounds were simulated using forcefields similar to those of the protein and then synthesized as ligands. Docking processes were carried out utilizing the C-Docker protocol within Discovery Studio 4.0, with the identical forcefields used previously. The interaction energies were sorted and recorded, along with the binding modes of the compounds, then displayed using 2D view modes.

### Standard dynamic simulations

Discovery Studio V. 4.0 was used for the dynamic simulations of free protein and rhamnolipid molecules. Standard Dynamic Cascades were used, with the initial minimization algorithm set to the steepest descent, maximum steps 2000, and RMS gradient 1.0. The second minimization algorithm was configured to use conjugate gradients with a maximum number of steps of 2,000. The initial temperature was set to 50, and the target temperature was 300, with a maximum velocity of 2000. In contrast, the equilibration phase was set with a simulation time of 200 ns. The Implicit Solvent Model was set to Generalised Born with Simple Switching (GBSW), and the dynamics integrator protocol was LeapfroyVerlet [17].

## Results and discussion

Inflammation is a complicated case of physiological changes caused by external causes that presents considerable hurdles in terms of treatment. Due to the rise of drug resistance and negative responses to synthetic anti-inflammatory medications, efforts have commenced to explore herbal remedies known for their rich resources, minimal side effects, and positive treatment outcomes. The advancement of natural anti-inflammatory treatments has become a point of research interest [9, 18–20].

### COX-1, 2, and 5-LOX inhibition assays

All this was already mentioned earlier-delete Evaluation of the potential COX inhibitory effect of the crude extract of the crude leaf extract of *D. erecta* and its fractions showed that the crude extract and the methylene chloride and ethyl acetate fractions had a COX inhibitory effect, with a strong preference for COX-2 (Table 1). Bioactivity-guided isolation yielded Duranterectoside A (**1**) from both bioactive fractions, suggesting that it may be the primary component responsible for the observed bioactivity in the plant. Not enough evidence to support this; it might just be a matter of polarity of the metabolite.

**Table 1:** IC_50_ values of methanolic extract and its respective fractions of *D. Erecta* Linn on COX-1, 2, and 5-LOX enzymes lettering of extract and fractions DO NOT coincide with those in the experimental section

Samples	COX-1	COX-2	5-LOX	SI
	IC_50_ Value (µg/mL)			
TE	1.8 ± 0.1	0.2 ± 0.05	7.7 ± 0.5	9.00
H-F	NA	NA	NA	-
MC-F	0.7 ± 0.01	0.09 ± 0.001	4.8 ± 0.4	7.78
ET-F	0.4 ± 0.03	0.05 ± 0.005	2.9 ± 0.1	8.00
BU-F	1.5 ± 0.05	0.1 ± 0.03	5.5 ± 0.4	15.00
Celecoxib	0.5 ± 0.02	0.07 ± 0.002	**-**	7.14
Zileuton	-	-	6.1 ± 0.2	-

**TE**: total methanolic extract; **H-F**: *n*-hexane fraction; **MC**: methylene chloride; **ET-F**: ethyl acetate fraction, **BU-F**: *n*-butanol fraction; **NA**: No activity; **SI**: Selectivity index, IC_50_ for COX-1/IC_50_ for COX-2

### Spectral data of the isolated compounds

**Fig. 1:**
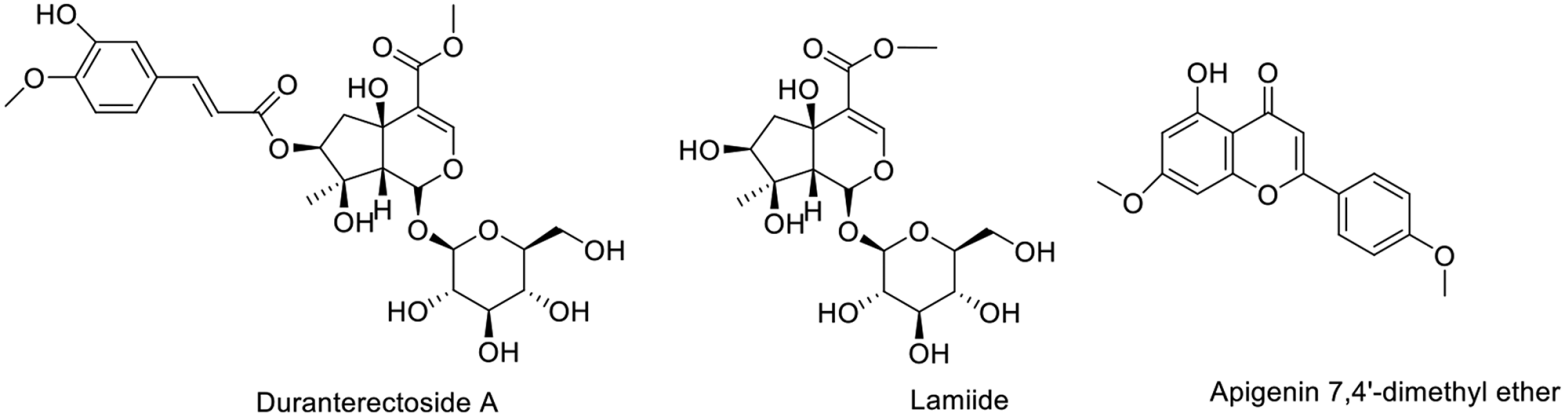
Structures of isolated compounds. Numbers should be included, in parenthesis, next to the names

The phytochemical analysis of the TE using various chromatographic techniques resulted in the separation of three compounds (compounds **1**–**3**) (Fig. 1 and Table S1). The isolated compounds were identified as two iridoids glycosides and one flavone aglycone that were identified based on their structures using spectroscopic data (ESI-MS, ^1^H-NMR, and ^13^C-NMR), as well as comparisons to published data (Figs. S2–S10).

Compound **2** was isolated as white powder. The molecular formula of compound **2** was determined by negative ion ESI-MS: *m/z* 421.2350 [M − H]^−^ for C_17_H_25_O_12_ and 467.24320 ([M + HCOO]^−^ for C_18_H_27_O_14_, in agreement with 17 observed resonances in the ^13^C-NMR spectrum (Table S1 and Figs. S2–S4). The ^1^H-NMR spectrum of **2** (Table S1, Fig. S3) displayed δ_H_ 7.46 (1H, s, H-3); 3.758 (s, MeOOC), a CH_2_ group (δ_H_ 2.32 (1H, dd, *J* = 16.1 and 1.7 Hz, H-6a) and 2.35 (1H, dd, *J* = 16.1 and 5.0 Hz, H-6b); an oxymethine (δ_H_ 4.77 (1H, dd, J = 5.0 and 1.7 Hz, H-7); and a Me group (δ_H_ 1.06 (3 H, s, H-10), a characteristic signals for a C-10 iridoid. The anomeric sugar proton at δ_H_ 4.56 (1H, d, *J* = 7.9 Hz, H-1‵), indicated the presence of a *β*-glucose unit. Both the chemical shifts and coupling constants of H_2_-6 and H-7 of **2** suggested that the 7-OH group was *β*-oriented. From these data (Table S1), the structure of compound **2** was identified as lamiide [21, 22].

Compound **1** was isolated as white powder, and determined through negative ESI-MS (*m/z* 597.3323 [M − H]^−^ for C_27_H_33_O_15_). The ^1^H and ^13^CNMR spectra of **1** were closely resembled to those of lamiide (**2**), with the exception of a downfield shift (ca. 1.49 ppm) for H-7, which appeared at 4.77 in 1, along with a downfield shift (ca. 3.4 ppm) for C-7, together with the upfield shifts for C-6 and C-8, indicating the presence of an acylated oxygen at C-7 in **1**. Moreover, signals indicative of a trans-isoferuloyl group were detected at C-7. Hence, it was confirmed that duranterectoside A (**1**) is the 7-*O*-trans-isoferuloyl ester of lamiide [23].

Compound **3** was isolated as yellow powder, and the molecular formula was determined through negative ion ESI-MS, showing *m/z* 297.2365 [M − H]^−^ for C_17_H_13_O_5_. In the ^1^H NMR spectrum of **3**, two singlets were observed at δ 3.63 (3H, s) and 3.75 (3H, s), attributed to the two methoxy groups at positions 4’ and 7’, respectively. Additionally, signals were detected at δ 6.42 (1H, s, H-3), two proton doublets at δ 7.69 (2 H, dd, J = 7.7, 2.2 Hz) and 7.09 (2 H, dd, *J* = 7.7, 2.2 Hz), corresponding to H2’/H6’ and H3’/H5’ protons. Two doublets at δ 6.28 (1H, brs) and 6.35 (1H, brs) were assigned to protons H-6 and H-8, respectively. In the ^13^C NMR spectrum of **3**, 15 signals were observed for 17 carbons. A signal at δ 182.37 was designated for C-4, while signals at δ 56.93 and 59.63 were linked to two methoxy groups at C-4 and C-7. Furthermore, signals at δ 127.95 and 115.65 were assigned to carbons C-2/6 and C-3/5, respectively. Signals at δ 101.16 and 94.18 were attributed to C-6 and C-8. Based on the spectral data provided, compound **3** was identified as apigenin 7,4’-dimethyl ether [24].

### In-vitro anti-inflammatory assays

Two iridoids, duranterectoside A **(1)**, lamiide **(2)**, and apigenin 7,4’-dimethyl ether **(3)**, were tested against the three enzymes (Table 2). Iridoids are the primary bioactive principles in *Duranta* spp., with potent pharmacological and biological activities such as anti-inflammatory, antioxidant, neuroprotective, cardioprotective, antitumor, hypoglycemic, hypolipidemic, antiallergic, antimalarial, antibacterial, antiviral, and insect repellent properties [25]. Lamiide (**2**) has been previously reported to have anti-inflammatory properties in carrageenan-induced rat paw edema and rat brain phospholipid tests, suggesting that **2** may exert its anti-inflammatory action by scavenging free radicals from the lipid membrane [26]. Similarly, it has been reported that lamiide (**2**) suppresses soybean 5-LOX, when tested in vitro [21] and that possesses a moderate inhibition of lipoxygenase at a dose of 0.5 mM [27]. Collectively, iridoids are reported to have anti-inflammatory activity by inhibition of phospholipid peroxidation, leukocyte accumulation and influx, free radical scavenging activity, and their inhibitory power against 5-lipooxgenase enzyme, in addition to histamine and bradykinin release [22]. Apigenin 4’,7-dimethyl ether, a methylated flavone, has been shown to have strong anti-inflammatory properties mainly due to its prostaglandin inhibitory potential [7]. Our findings indicate that the three isolated compounds exhibit anti-inflammatory activity, primarily mediated by their selective inhibition of COX-2, along with potential inhibitory effects on 5-LOX.

**Table 2:** IC_50_ values of the isolated compounds (1–3) on COX-1, -2, and 5-LOX enzymes

Samples	COX-1	COX-2	LOX	SI
	IC_50_ Value (µM)			
Compound **1**	7.3 ± 0.9	0.08 ± 0.001	2.6 ± 0.1	91.25
Compound **2**	8.5 ± 0.2	0.1 ± 0	4.9 ± 0.4	85.00
Compound **3**	9.9 ± 0.6	0.29 ± 0.02	3.9 ± 0.6	34.14
**Celecoxib**	14.8 ± 0.2	0.045 ± 0.002	**-**	328.89
**Zileuton**	-	-	3.5 ± 0.2	-

Compound **1**: duranterectoside A; compound **2**: lamiide; compound **3**: apigeninn 4’, 7-dimethyl ether; **SI**: Selectivity index: IC_50_ for COX-1 / IC_50_ for COX-2

### In silico studies

Discovery Studio 4.0 software was used for in silico molecular docking, dynamic modelling, and ADMET experiments.

### Molecular docking

**Fig. 2:**
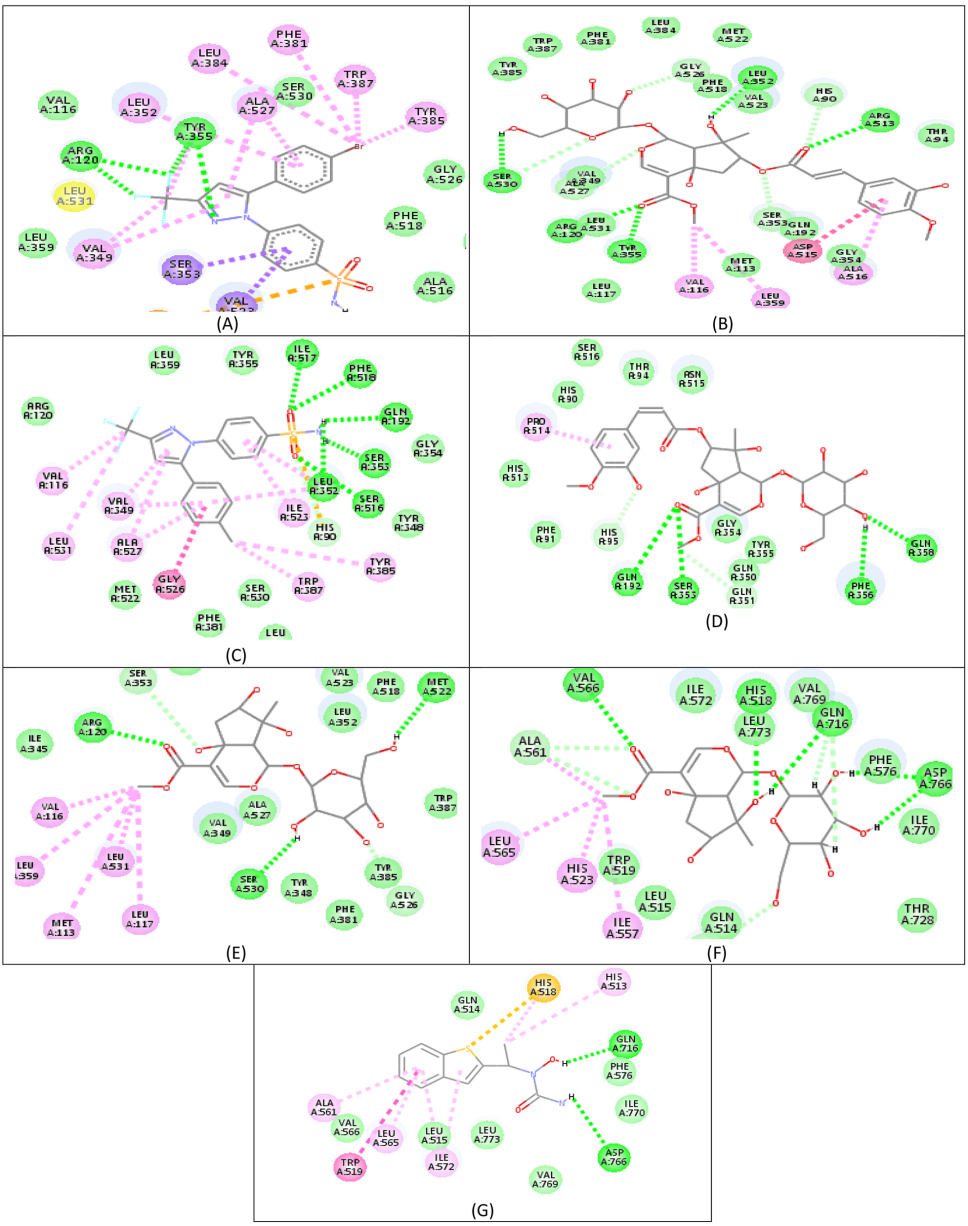
**(A**,** C)** Retrieved docking pose of Celecoxib with COX-2 and COX-1, respectively showing the key interactions as reported. (**B**, **D**) Docking poses of compound **1** with COX-2 and COX-1, respectively. (**E**,** F**) docking pose of compound **2** with COX-2 and LOX, respectively, (**G**) docking pose of Zileuton with LOX

Molecular docking studies are a powerful method for understanding the various interactions between ligands and the active sites of enzyme. The in-vitro anti-inflammatory activity of isolated compounds **1**, **2**, and **3** against COX-1, 2, and LOX prompted us to conduct a molecular docking study to interpret the biological results and gain a better understanding of the binding poses and interactions with the key amino acids in each enzyme’s binding site. Celecoxib was co-crystallized with COX-1 and COX-2, which were obtained from the protein data bank (PDB ID: 3KK6) [14] and (PDB ID: 1CX2) [15] and gallocatechin co-crystallized with the LOX (PDB ID: 1JNQ) [16] were used as reference compounds to evaluate the molecular modeling docking study results. To validate the C-Docker methodology used in this investigation, the lead chemical was re-docked into the active sites of each enzyme. The results showed good agreement, with RMSD values of 0.5 Å, 0.4 Å, and 6 Å for COX-1, COX-2, and 5-LOX, respectively. These results support the efficacy of the docking approach used [28]. The presented docking study revealed only comparable binding mechanisms between the lead drug and the docked molecules, particularly for COX-2. Table 3 summarizes the CDOKER-interaction energy and the drugs’ interactions with critical amino acids via COX-2. The molecular docking of the three compounds using the C-Docker methodology revealed that compounds 1 kept two essential H-bonds with TYR355 and ARG120 in COX-2 and one essential H-bond with GLN192 in COX-1 active site compared to the reference compound celecoxib (Fig. 2). It is worth noting that the phenylacryloyl moiety of compound 1 enhanced its interaction with COX-2 and LOX, where it revealed a hydrogen bond with Arg513 and carbonyl oxygen of COX-2. In addition, the substituted phenyl ring lay in a deep extended hydrophobic pocket through hydrophobic interaction with Asp515 and Gly354. Furthermore, Compound **1** demonstrated greater docking scores than the docked lead compounds. These findings helped to explain why compound **1** had such a strong inhibitory effect when compared to the other derivatives.

**Table 3:** The C-Docker interaction energy and key amino acids interaction for COX-2

Compound NO	CDOCKER interaction energy (kcal/mol)			Key amino acids for interactions with COX-2	
	COX-1	COX-2	LOX		
Compound **1**	44.1	55.5	38.5	H-Bond	HB-Distance (Ǻ)
				TYR355 ARG120 LEU352 SER530 ARG513	2.28 2.75 2.81 2.64 3.01
Compound **2**	40.7	51.2	33.6	ARG120 SER530 MET522	2.84 2.66 2.48
Compound **3**	39.8	46.8	34.5	TYR355 LEU325	2.55 2.94
**Lead comp. 1 (celecoxib)**	37.9	55	-	ARG120 ARG120 TYR355 TYR355	2.45 2.73 2.12 2.88
**Lead comp. 2 (gallocatechin**)	-	-	37.9		-
**Zileuton**	-	-	36.98	ASP766 GLN716	3.12 2.79

### Standard dynamic simulation

Molecular dynamic simulations were carried out to investigate the ligand-protein interaction in motion that contributes to their stable bound conformation and to visualize the influence of ligand binding on protein conformational changes [10]. To better understand the action of compound **1** on COX-2, MD simulations were done first on the free protein COX-2 (without any ligand), followed by MD simulations of COX-2 in contact with compound **1**.

**Fig. 3:**
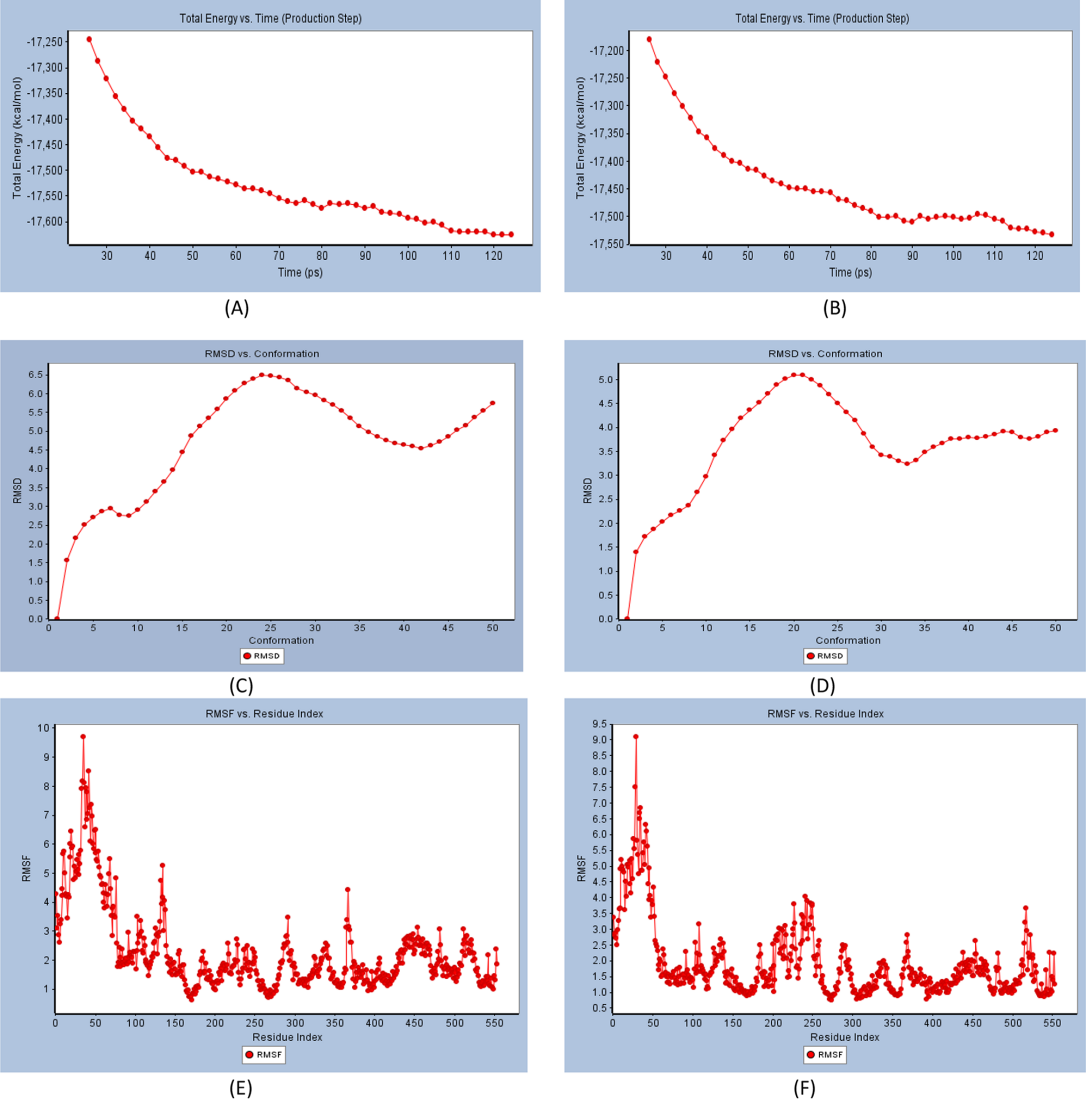
**(A)** Total energy Vs time in production step of free protein, (**B**) Total energy *Vs* time in production step during interaction of compound **1** best conformation poses with protein COX-2. (**C**) RMSD for free protein (**D**) RMSD for compound **1**-COX-2 complex. (**E**) RMSF for free protein. (**F**) RMSF for compound **1**-COX-2 complex

**Fig. 4:**
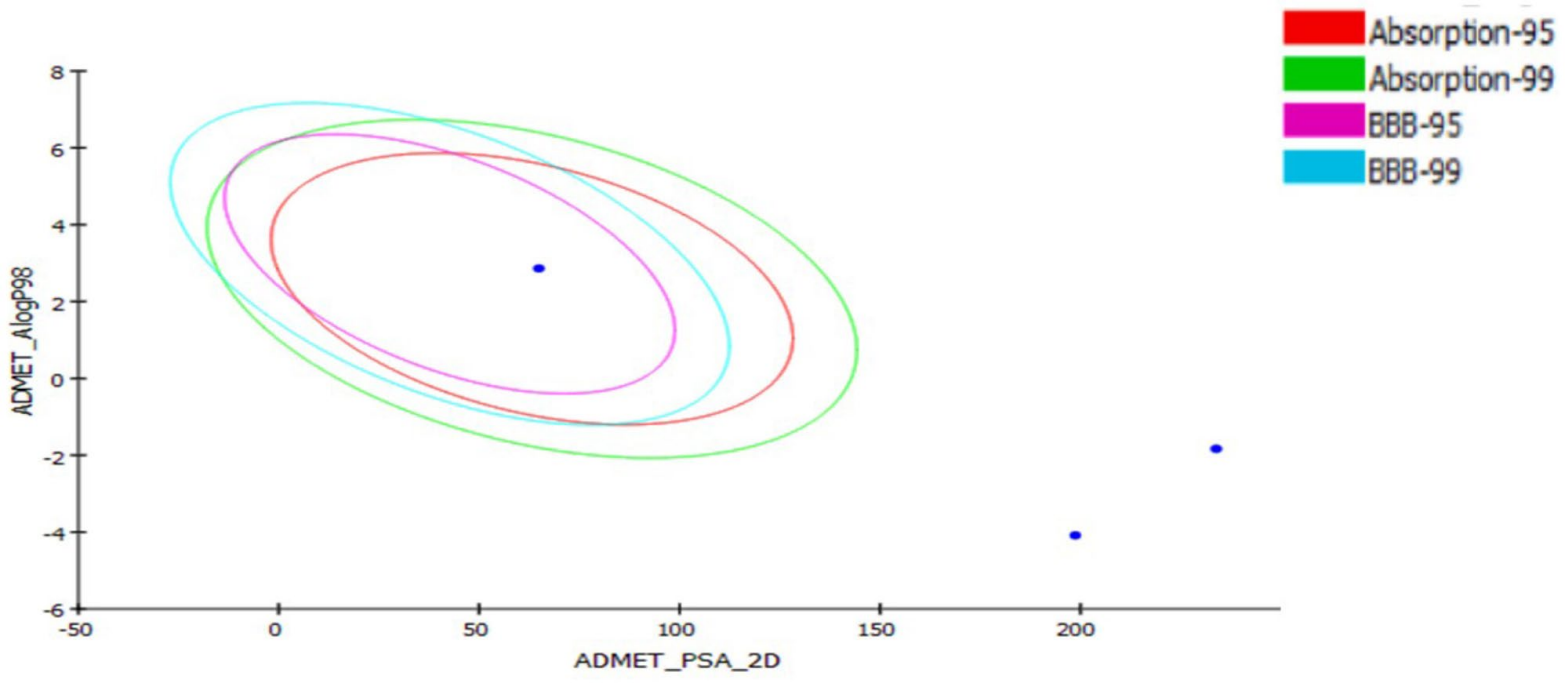
The ADMET plot uses calculated PSA_2D and A log P98 properties

The RMSD (Fig. 3c), root mean square fluctuations (RMSF) (Fig. 3e), and energy (Fig. 3a) were investigated for free Protein COX-2, and their contributions were plotted as a time-dependent function of MD simulations. The RMSD of the protein backbone was found to fluctuate around 4Å (Fig. 3c). These averaged constant graphs indicate that the protein structure remains steady during each 200 ns simulated time period. The RMSF of all residues was determined during the 200 ns MD simulations to identify the protein’s higher flexibility areas. In COX-2 RMSF graph (Fig. 3e), significant peaks of fluctuations have been seen with the initial 50 residues with over 9.8 Å, residue number 120 up to 5.6 Å, residue 360 with 4.5 Å, and between 400 and 550 with up to 3.0 Å. Finally, we have analyzed the energy involved for the stabilized conformation of this protein and it was observed to have an averaged − 17,250 and − 17,600 kcal/mol of energy (Fig. 3a). Taking all of the foregoing observations in RMSD, RMSF, and energy contributions into account, it is possible to conclude that the COX-2 protein structure remains rather stable throughout the simulation. MD simulations of COX-2 in complex with compound **1** were carried out to better understand the effect of compound **1** binding to COX-2. MD simulations were conducted using the best docked pose of the COX-2-compound **1** binding complex with a binding energy of − 55.5 kcal/mol obtained from protein-ligand docking (Fig. 2). The results are given on Fig. 3. Figure 4d shows that the RMSD of the trajectories for COX-2 in complex with 1 fluctuates around 3.0 Å.

These findings strongly indicate compound **1**’s substantial inhibitory and stabilizing capabilities on COX-2 when compared to COX-2 residue variations in the absence of ligand, which were found to exhibit highly fluctuating peaks as described in the preceding section. The 200 ns simulation time used in this investigation is sufficient to allow for side chain rearrangements in both the natural and protein-compound **1** complexes, resulting in the most stable binding configuration [15]. In addition, RMSF was determined for each residue index to assess the flexibility of the COX-2-compound **1** complex, which exhibited a significant decrease in fluctuations compared to the free protein, indicating improved stability (Fig. 3f). After analyzing the total energy involved for the stabilized conformation of this protein in complex with 1, we found that it maintains an average of − 17,200 and − 17,550 kcal/mol of energy (Fig. 3b), which is well minimized in contrast to COX-2 in the absence of ligand.

### In silico predictive ADMET study

The ADMET study focuses on the chemical structure of the molecule and includes the calculation of several parameters using Discovery Studio 4.0 software: blood-brain barrier level, absorption level, atom-based log P98 (A log P98), 2D polar surface area (ADMET 2D PSA), Cytochrome P450 2D6 (CYP2D6), hepatotoxicity probability, aqueous solubility level, and plasma protein binding logarithmic level (PPB Level) (Fig. 4; Table 4). Compounds **1** and **2** in the ADMET plot had BBB levels of 4, indicating that they cannot breach the blood-brain barrier and so will not induce CNS adverse effects. While compound **3** can pass through the BBB. Compounds **1** and **2** are expected to have limited intestinal absorption, but compound **3** demonstrated high absorption (Fig. 4). All of the substances had an ADME aqueous solubility level between 3 and 4, indicating acceptable solubility. The CYP2D6 score indicates whether a specific chemical structure inhibits or does not inhibit the Cytochrome P450 2D6 enzyme. Compounds **1** and **2** are predicted to be non-inhibitors of CYP2D6, hence side effects such as liver dysfunction are not expected with their administration, as opposed to compound **3**. The plasma protein-binding parameter indicates the degree to which a drug binds to carrier proteins. These chemicals can be anticipated to reach the desired targets because they all showed 90% plasma protein binding. The main property (PSA) is a determinant influencing medication bioavailability. Thus, passively absorbed compounds with PSA levels greater than 140 are assumed to have low bioavailability. Compounds **1** and **2** have PSA values greater than 140, indicating limited passive oral absorption, with the exception of compound **3**, which has a PSA value of 64.906 and good bioavailability.

**Table 4:** ADMET predictions of the compounds 1, 2 and 3

Cpd	Log P98	ADMET Absorption level	BBB level	ADMET PSA 2D	Cytochrome P450 2D6 (CYP2D6)	Hepato-toxicity	DMET Aq solubility level	PPB-level
1	-1.833	3(v. Low)	4	233.89	-7.89(non-inhibitor)	false	3(Good)	16.3141
2	-4.086	3 (v. Low)	4	198.729	-3.77(non-inhibitor)	false	4(Good)	14.483
3	2.861	0 (Good)	2 (Pass)	64.906	-0.98	true	3(Good)	10.8184

## Conclusion

To summarize, the plant extract has a notable dual inhibitory effect on 5-LOX and COX-2. This dual activity has the potential to improve its anti-inflammatory activities while also reducing the gastrointestinal side effects often associated with nonsteroidal anti-inflammatory medications (NSAIDs). However, more research is needed to properly analyze the anti-inflammatory potential of compounds **1** and **2**, including in vivo efficacy trials and pharmacokinetics assessments. The compounds showed promising bioavailability and no CNS negative effects. As a result, these found compounds will aid in the development of innovative and promising COX-2 inhibitors that are both alternative and effective. Future research will shed light on the compound’s efficacy and potential therapeutic option for inflammation-related conditions such as controlling pulmonary inflammation or as potential anti-arthritic agents.

## Electronic supplementary material

Below is the link to the electronic supplementary material.

Supplementary Material 1

## Data Availability

Not applicable (this manuscript does not report data generation or analysis).
