# Supplementary material for: Bioactivity-guided isolation and molecular modeling of the anti-inflammatory constituents from the leaves of Duranta erecta Linn
Source: BMC Complement Med Ther. 2025 Jan 28;25:31. doi: 10.1186/s12906-025-04764-7 (PMC11773759; doi:10.1186/s12906-025-04764-7)
Supplement: Supplementary file 1 — Supplementary Material 1 [file 12906_2025_4764_MOESM1_ESM.pdf]

Table S1. Spectral data of isolated compounds

Compound No.	Data	Ref.
**1**	^{1}H-NMR (400 MHz, CD_3OD): *δ* (ppm); 1.06 (3H, s, H-10), 2.32 (1H, dd, J = 16.1 and 1.7 Hz, H-6), 2.35 (1H, dd, J = 16.1 and 5.0 Hz, H-6), 2.86 (1H, s, H-9), 3.78 (3H, s, COOMe), 3.81 (3H, s, OMe), 4.56 (1H, d, J = 7.9 Hz, H-1\`), 4.77 (1H, dd, J = 5.0 and 1.7 Hz, H-7), 5.75 (1H, d, J = 0.7 Hz, H-1), 6.48 (1H, d, J = 15.9 Hz, H-8\`\`), 6.83 (1H, d, J = 8.3 Hz, H-5\`\`), 7.27 (dd, J = 8.3 and 1.9 Hz, H-6\`\`), 7.28 (1H, d, J= 1.9 Hz, H-2\`\`), 7.46 (1H, s, H-3) and 7.64 (1H, d, J= 15.9 Hz, H-7\`\`). $^{13}$CNMR (100 MHz, $CD_3OD$): δ (ppm); 92.75 (C-1), 151.01 (C-3), 115.14 (C-4), 67.79 (C-5), 44.41 (C-6), 79.43 (C7), 78.98 (C-8), 56.92 (C-9), 20.23 (C-10), 167.32 (C-11), 50.80 (COOMe), 98.33 (C-1\`), 73.02 (C-2\`), 78.98 (C-3\`), 70.25 (C-4\`), 77.55 (C-5\`), 61.43 (C-6\`), 129.78 (C-1\`\`), 113.23 (C-2\`\`), 151.01 (C-3\`\`), 145.28 (C-4\`\`), 114.20 (C-5\`\`), 119.31 (C-6\`\`), 145.14 (C-7\`\`), 166.89 (C-8\`\`), 168.71 (C-9\`\`), 56.92 (OMe). Negative ion ESI-MS: *m/z* 597.3323 $[M-H]^-$ for $C_{27}H_{33}O_{15}$, identified as duranterectoside A.	**[1]**
**2**	^{1}H-NMR (400 MHz, CD_3OD): δ (ppm); 5.71 (H-1, 1H, s), 7.30 (H-3, 1H, m), 2.68 (H-6, 2H, m), 2.12 (H-6, 2H, m), 3.28 (H-7, 1H, m), 2.68 (H-9, 1H, m), 0.98 (H-10, 1H, s), 3.63 (H-12, OCH_3, s), 4.50 (H-1\`, 1H, m), 2.86 (H-2\`, 1H, m), 3.18 (H-3\`, 1H, m), 3.04 (H-4\`, 1H, m), 3.13 (H-5\`, 1H, m), 3.71 (H-6\`, 2H, m), 3.30 (H-6\`, 2H, m). $^{13}$C-NMR (100 MHz, $CD_3OD$): δ (ppm); 93.07 (C-1), 150.84 (C-3), 114.48 (C-4), 67.57 (C-5), 45.37 (C-6), 76.04 (C-7), 77.27 (C-8), 56.99 (C-9), 19.98 (C-10), 166.51 (C-11), 50.38 (C-12), 98.23 (C-1\`), 73.07 (C-2\`), 76.97 (C-3\`), 70.29 (C-4\`), 77.73 (C-5\`), 61.41 (C-6\`). Negative ion ESI-MS: *m/z* 421.2350 $[M-H]^-$ for $C_{17}H_{26}O_{12}$, identified as lamiide.	**[2]**
**3**	^{1}H-NMR (400 MHz, CD_3OD): δ ppm 6.28 (1 H, brs, H-8); 6.35 (1H, brs, H-6); 6.42 (1H, s, H-3); 7.09 (2H, dd, *J*=7.7, 2.2 Hz, H-3' & H-5'); 7.69 (2H, dd, *J*=7.7, 2.2 Hz, H-2'and H-6'); 3.63 (3H, s, 4'-OCH_3); 3.75 (3H, s, 7-OCH_3). ^{13}C-NMR (100 MHz, CD_3OD): δ ppm 94.18 (C-8), 101.16 (C-6), 102.15 (C-3), 103.06 (C-10), 115.65 (C-3' & C-5'), 121.18 (C-1'), 127.95 (C-2' & C-6'); 161.43 (C-9), 158.12 (C-5), 161.43 (C-2), 164.44 (C-4'), 166.67 (C-7), 182.37 (C-4), 56.93 (4'-OCH3), 59.63 (7-OCH3). Negative ion ESI-MS: *m/z* 297.2365 $[M-H]^-$ for $C_{17}H_{13}O_5$, identified as apigenin 7,4'-dimethyl ether.	**[3]**

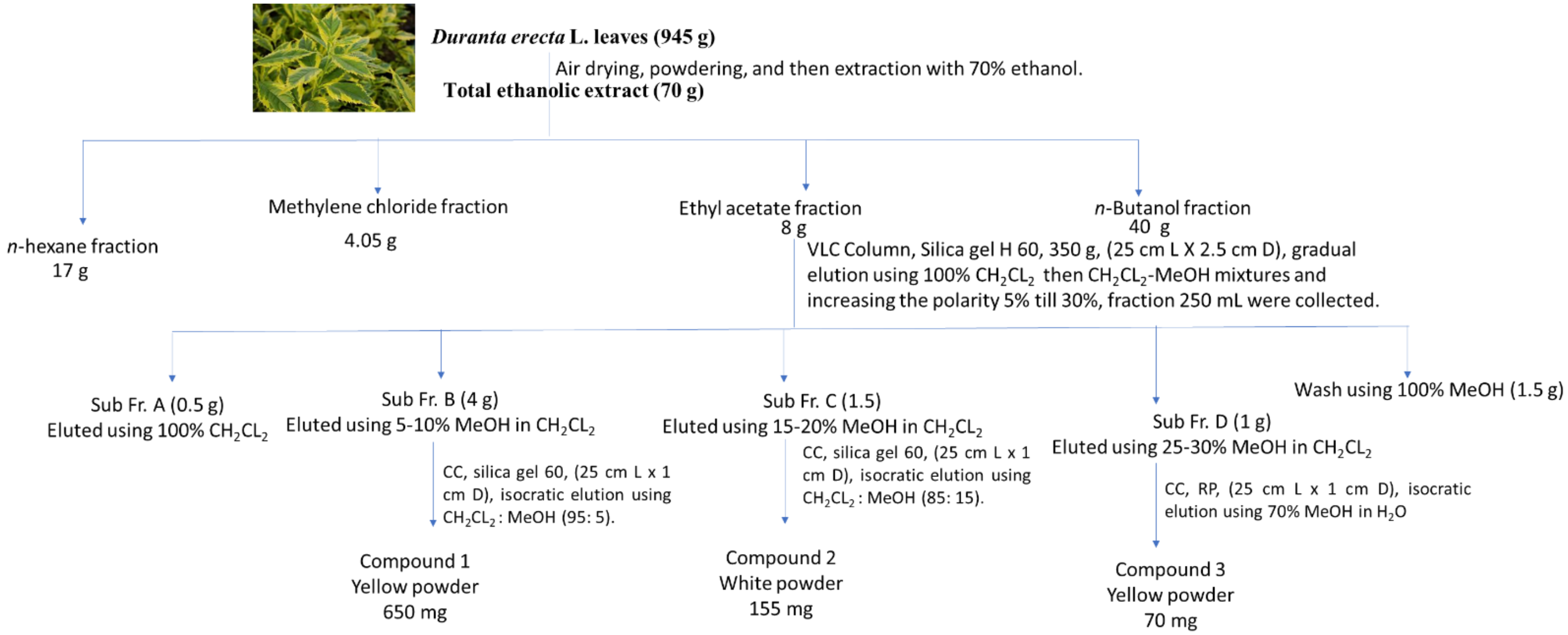

Figure S1. Chromatographic isolation from the bioactive ethyl acetate fraction of *D. erecta* L.

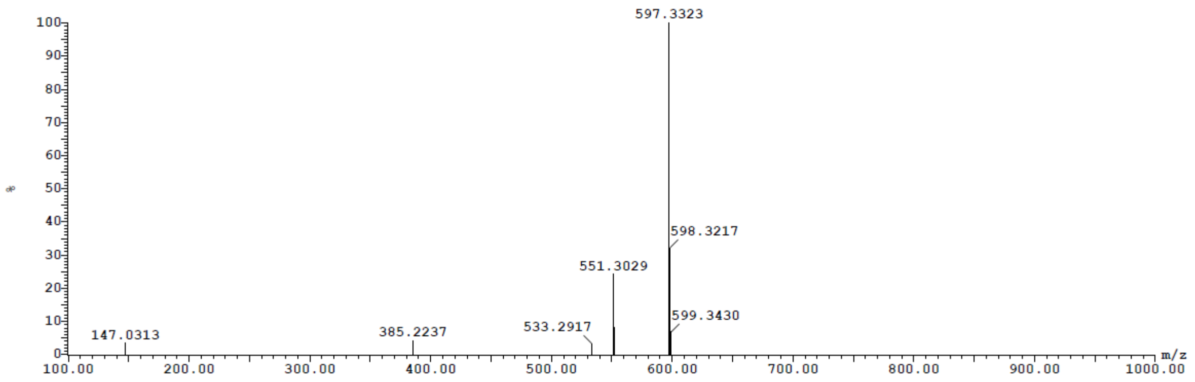

Figure S2. ESI-MS of Compound 1.

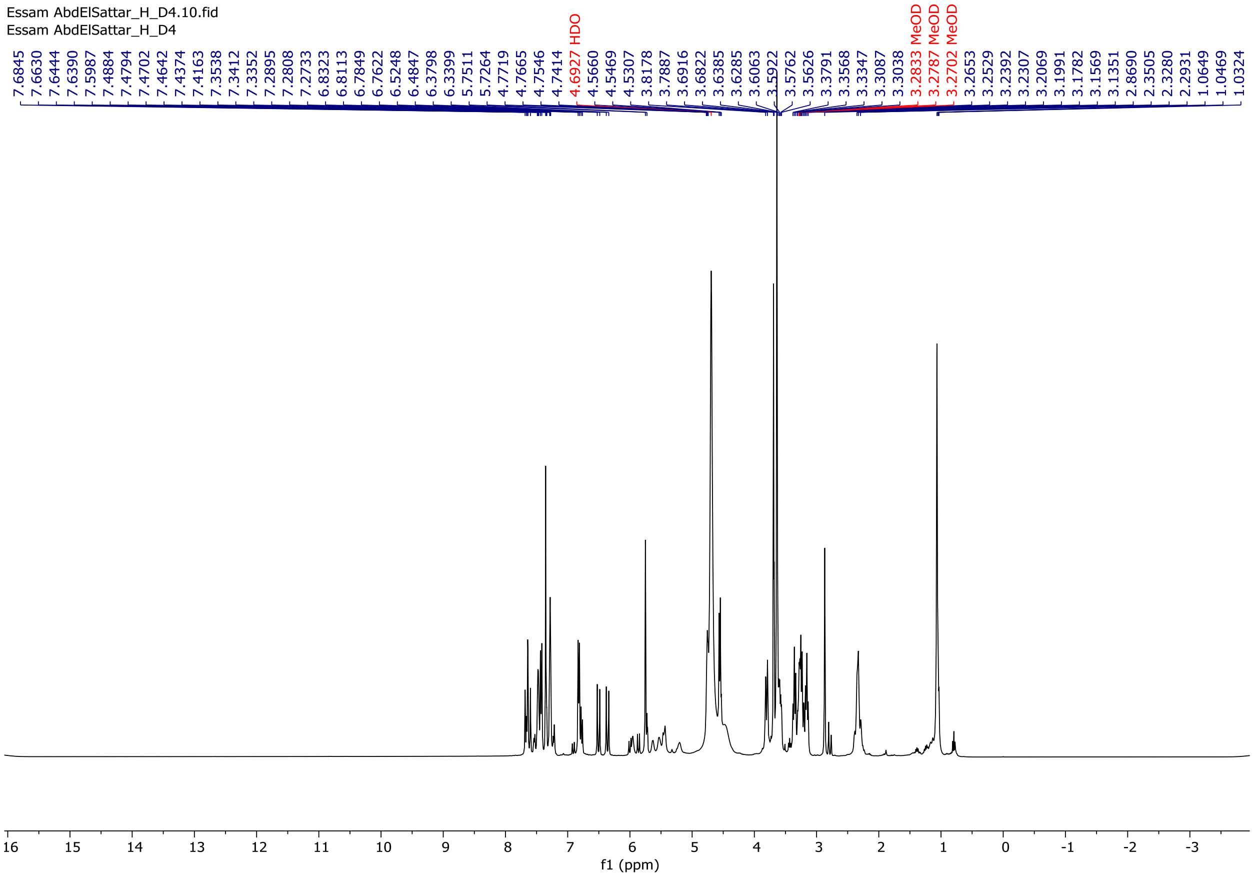

Figure S3. ^{1}HNMR of Compound 1.

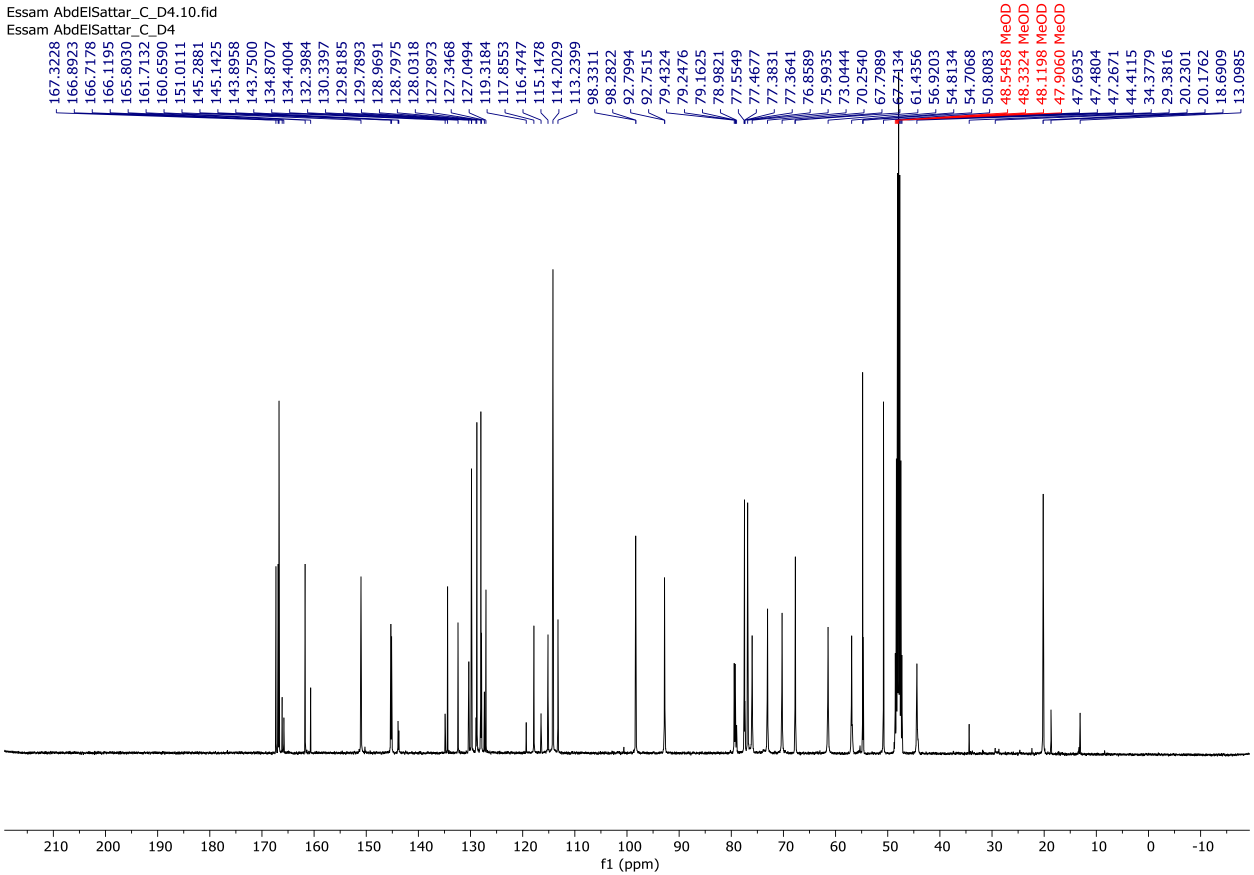

Figure S4. ^{13}CNMR of Compound 1.

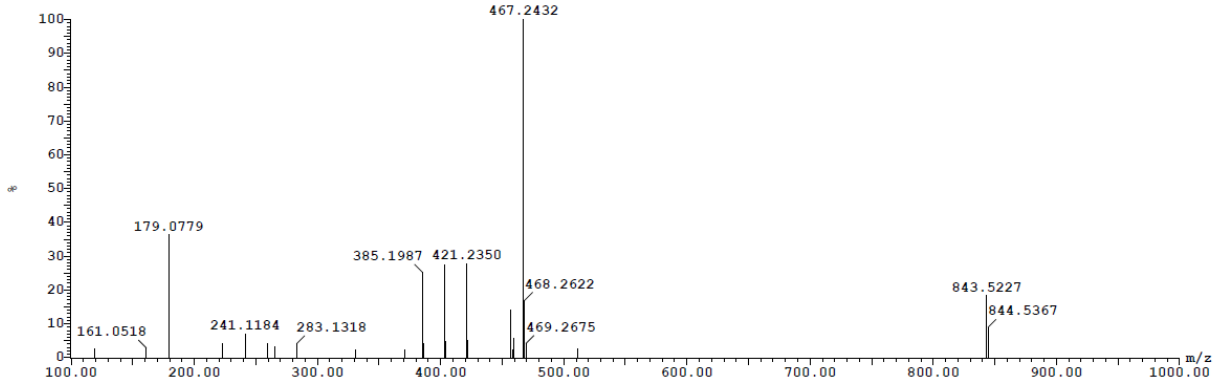

Figure 5. ESI-MS of Compound 2.

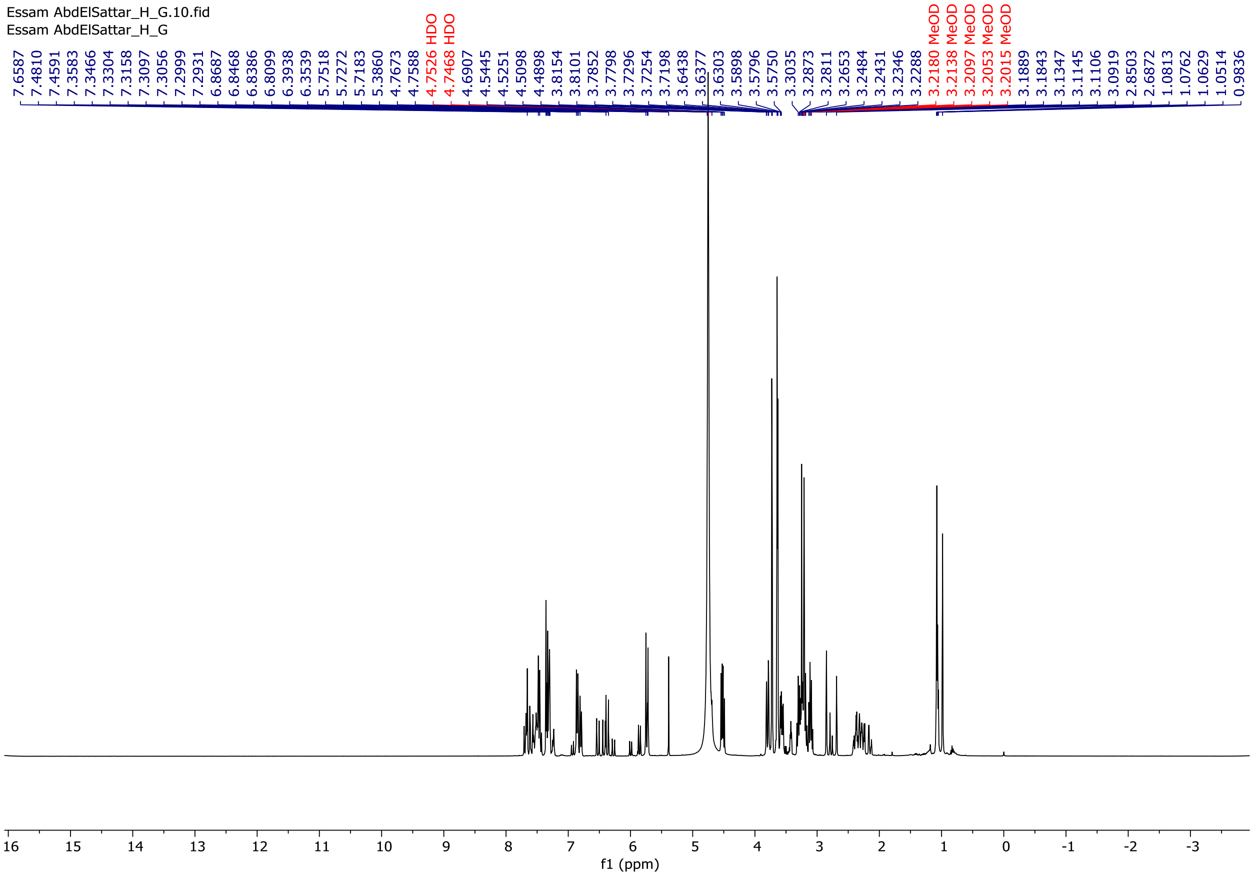

Figure S6. ^{1}HNMR of Compound 2.

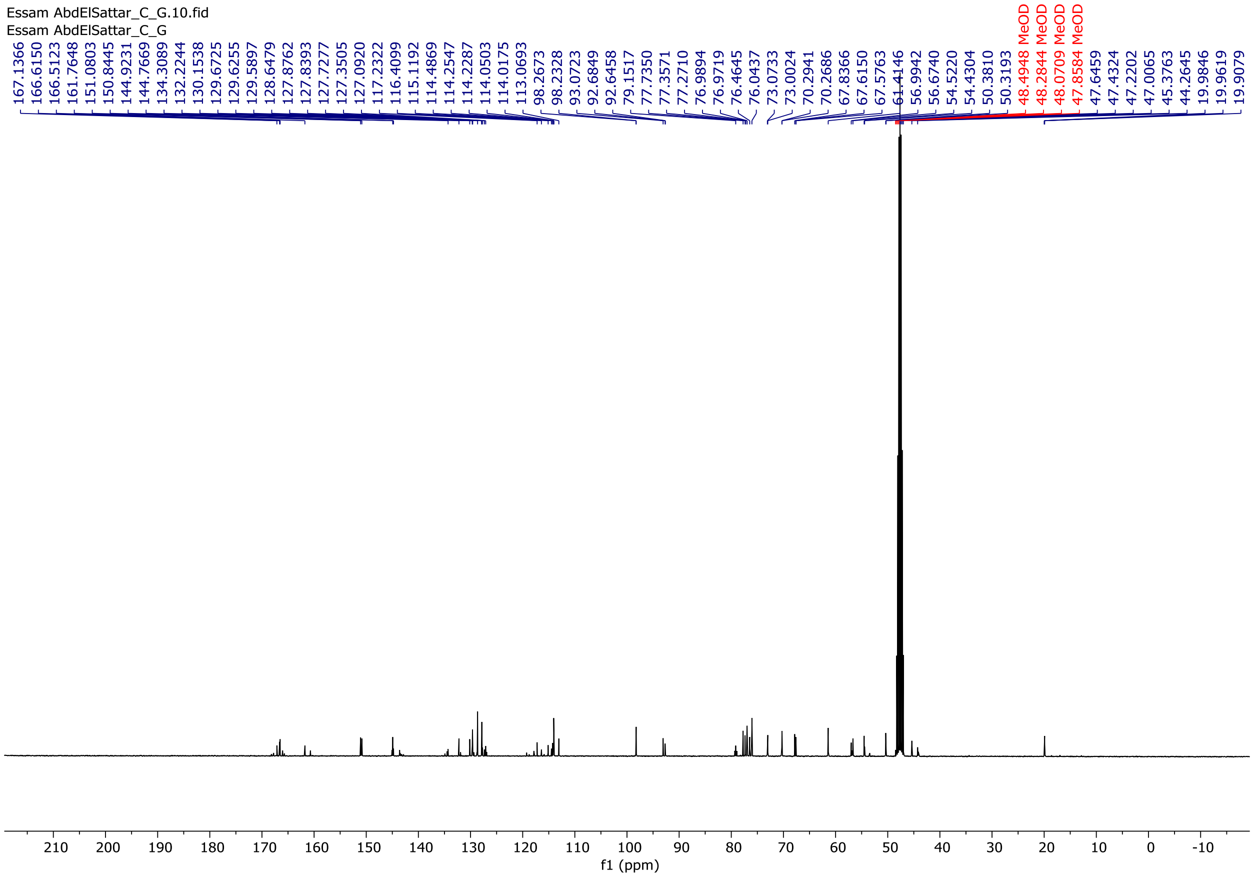

Figure S7. ^{13}CNMR of Compound 2.

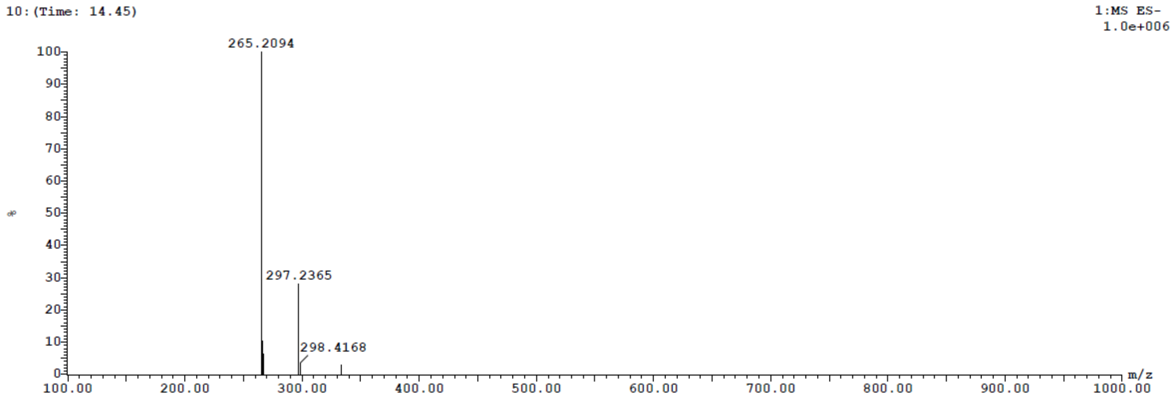

Figure S8. ESI-MS of Compound 3.

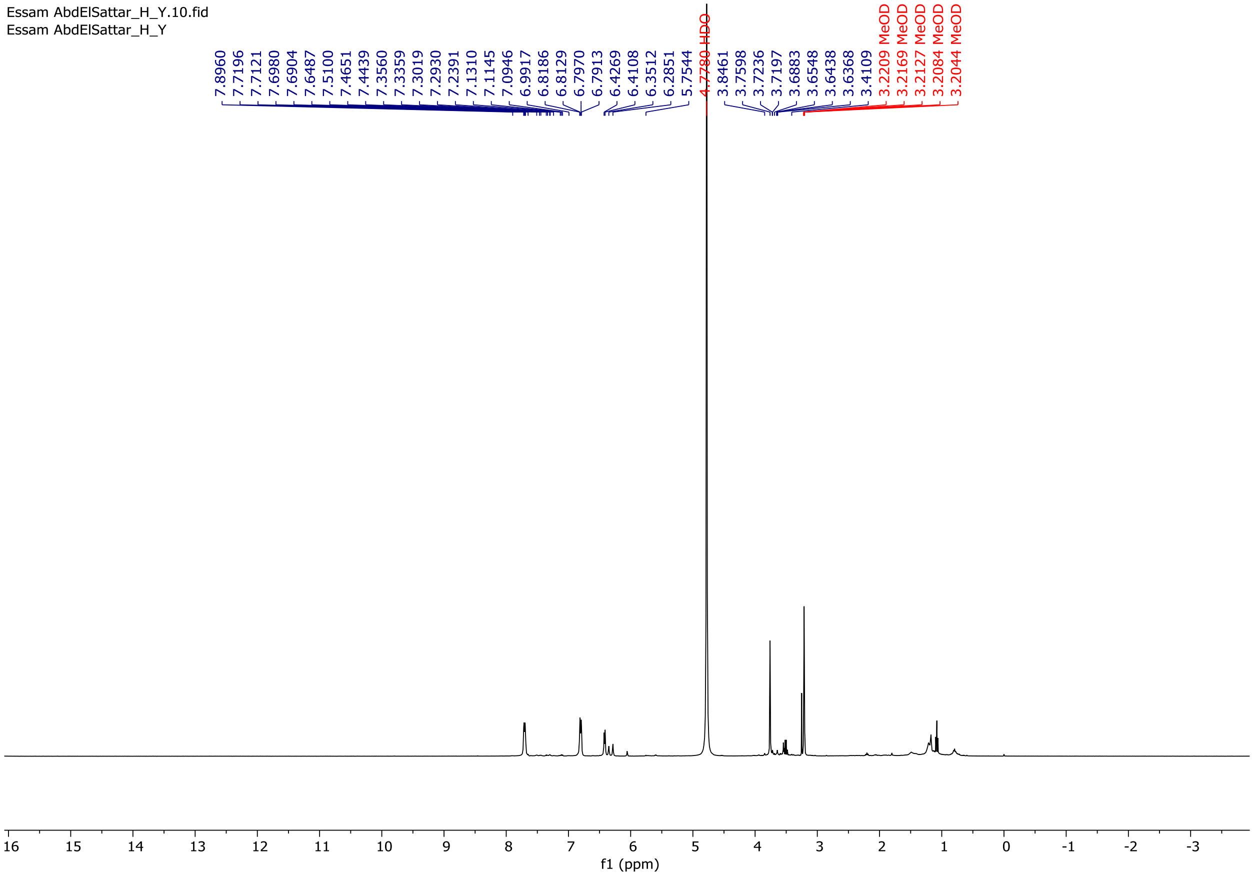

Figure S9. ^{1}HNMR of Compound 3.

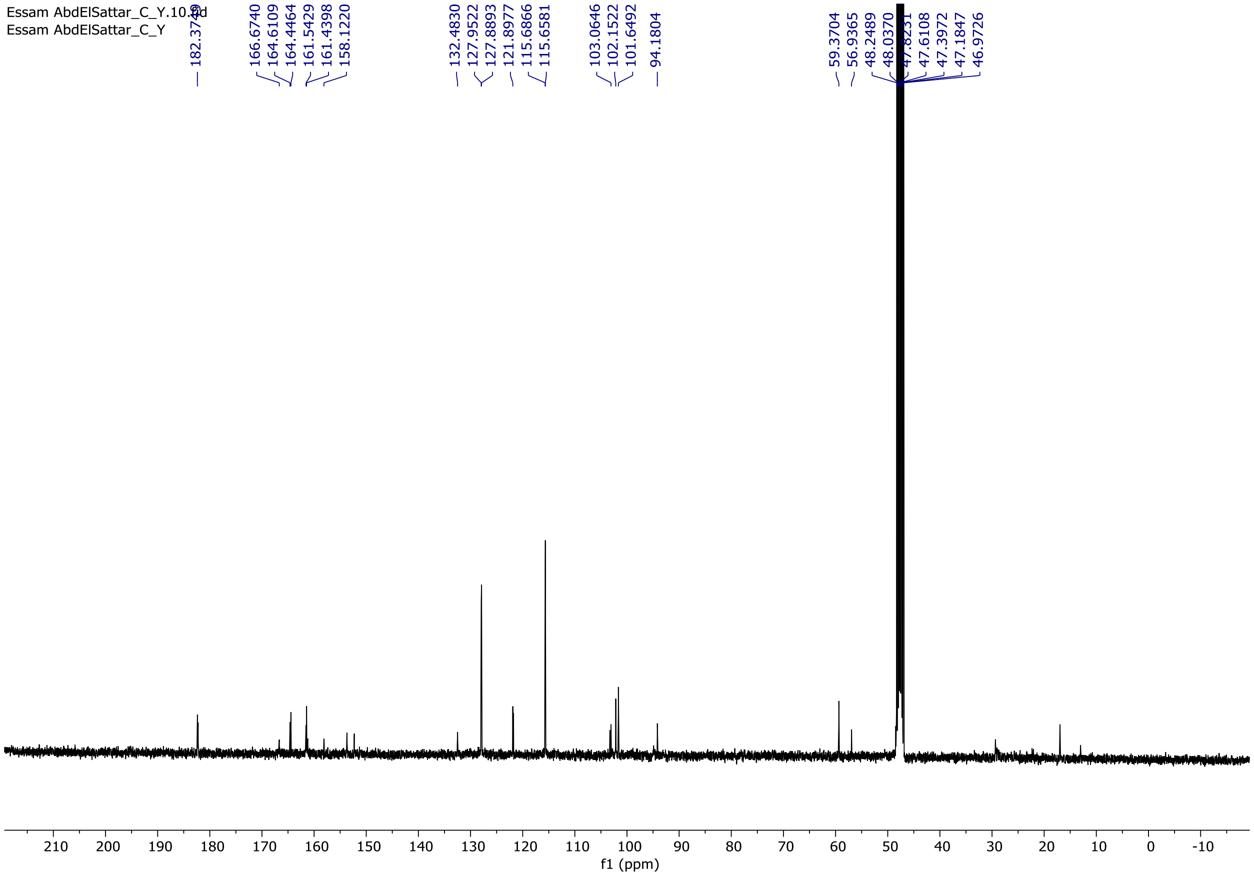

Figure S10. ^{13}CNMR of Compound 3.

[1] Takeda Y, Morimoto Y, Matsumoto T, Ogimi C, Hirata E, Takushi A, et al. Iridoid glucosides from the leaves and stems of Duranta erecta. Phytochemistry 1995;39:829-33.
[2] Sobeh M, Mamadalieva NZ, Mohamed T, Krstin S, Youssef FS, Ashour ML, et al. Chemical profiling of Phlomis thapsoides (Lamiaceae) and in vitro testing of its biological activities. Medicinal Chemistry Research 2016;25:2304-15.
[3] Saraswathy A, Vidhya B, Amala K. Apigenin-4', 7-dimethyl ether from Aquilaria agallocha roxb. Indian drugs 2012;49:30-2.
